# Association of residual methane emission classifications with intake, growth performance, feed efficiency, feeding behavior, and ruminal fermentation in crossbred beef cattle fed a forage-based diet

**DOI:** 10.1093/tas/txag159

**Published:** 2026-10-10

**Authors:** Musah Muntari, Zachary Seekford, Thainá Minela, Matthew Beck, Mahlatsana Ramaesela Ledwaba, Gordon E Carstens, Ky G Pohler, G Cliff Lamb

**Affiliations:** Department of Animal Science, Texas A&M University, College Station, TX 77843, United States; Department of Animal Science, Texas A&M University, College Station, TX 77843, United States; Department of Animal Science, Texas A&M University, College Station, TX 77843, United States; Department of Animal Science, Texas A&M University, College Station, TX 77843, United States; Agricultural Research Council, Animal Production, Germplasm Conservation and Reproductive Biotechnologies, Pretoria, 0062, South Africa; Department of Animal Science, Texas A&M University, College Station, TX 77843, United States; Department of Animal Science, Texas A&M University, College Station, TX 77843, United States; Department of Animal Science, Texas A&M University, College Station, TX 77843, United States; Texas A&M AgriLife Research, College Station, TX 77843, United States

**Keywords:** residual methane emissions, beef cattle, efficiency, digestibility, eating behavior

## Abstract

Residual methane (CH_4_) emissions (RME) have been proposed as a trait to identify low-emitting ruminants independent of intake and body weight. There is limited understanding of the biological mechanisms underlying the variation in CH_4_ emissions in animals with similar body weight (BW) and growth rate. This study evaluated the effects of divergent RME classifications on dry matter intake (DMI), growth performance, feed efficiency, feeding behavior, enteric CH_4_ emissions, and ruminal fermentation in crossbred beef cattle fed a forage-based diet. Eighty crossbred beef cattle (19 steers, 61 heifers; 463 ± 57 kg initial BW; mean age 551 ± 22 days) were monitored for 84 d using automated feed intake bunks and a GreenFeed Emission Monitoring (GEM) system. Residual CH₄ emission was computed for individual animals as the residuals from a model regressing CH_4_ emission on DMI and metabolic BW. Animals were ranked and classified as low RME (RME < 0.5 SD below the mean, *n* = 25), medium RME (RME ± 0.5 SD above and below the mean, *n* = 27), or high RME (RME > 0.5 SD above the mean, *n* = 26). The RME classification did not affect (*P* ≥ 0.39) DMI, average daily gain (ADG), or metabolic BW. A tendency (*P* = 0.08) was observed towards a lower gain-to-feed ratio in low RME animals relative to medium and high RME counterparts. Feeding behavior traits, including bunk visit duration and eating rate, were unaffected (*P* ≥ 0.80) by RME, although low RME animals tended to have fewer bunk visits (*P* = 0.08). Enteric gas fluxes were markedly different across groups: low RME cattle produced 20% less CH₄ (163 vs. 205 g/d; *P* < 0.001), 20% less CH_4_ yield (17.9 vs. 22.4 g/kg DMI; *P* < 0.001), and 21% less CH_4_ per MBW (1.51 vs. 1.92 g/kg BW^0.75^; *P* < 0.001) compared with high RME animals. Carbon dioxide (CO_2_) production was 8% lower (*P* < 0.01) and H_2_ tended to be lower (*P* = 0.06) in the low RME group. Molar proportions of volatile fatty acids (VFAs) exhibited no differences between low and high RME cattle. Residual methane emission was positively correlated with CH_4_ production (r = 0.81), CH_4_ yield (r = 0.63), CH_4_ per MBW (r = 0.88), G:F (r = 0.26) and negatively correlated with RFI (r = −0.14). Collectively, these findings demonstrate that cattle divergently classified for low RME produce substantially less CH₄ without compromising ADG. Although a tendency toward reduced feed efficiency was observed, the biological relevance of this difference warrants further investigation, particularly given the diet-type and mixed-sex population evaluated. These results support RME as a biologically meaningful and applicable selection trait for CH₄ mitigation in beef production systems.

## Teaser

Our study shows that beef cattle classified as low residual CH₄ emitters released ∼20% less CH₄ without affecting growth, intake, however a tendency towards reduced feed efficiency which warrants further investigation. This reinforces RME as a selection tool to reduce CH_4_ emissions in beef cattle.

## Introduction

Enteric CH_4_ is a by-product of microbial fermentation of feed in the rumen and represents an energy loss equivalent to 2–12% of gross energy intake in ruminants (Johnson and Johnson 1995; Moss et al. 2000; Ungerfeld 2020). Methane is a short-lived but potent greenhouse gas with a global warming potential 27 times greater than carbon dioxide (CO₂) over 100 years (IPCC 2021). Livestock contributes approximately 14.5% of total global anthropogenic greenhouse gas emissions, with enteric fermentation alone accounting for about 44% of the livestock sector’s emissions (Gerber 2013). Mitigation of CH_4_ is widely recognized as a critical near-term strategy for climate stabilization given its relatively short atmospheric lifetime and high warming potential (Ocko et al. 2021; Beck et al. 2023). Implementing CH₄ mitigation measures in ruminant systems may enhance production efficiency while contributing to global climate sustainability goals.

Variations in enteric CH₄ production among individual animals have been attributed to differences in feed intake, digestive efficiency, and the composition of the rumen microbiome (Pinares-Patiño et al. 2003; Pitta et al. 2022). While CH₄ production (g/d) is often positively correlated with dry matter intake (DMI) and animal size, CH₄ yield (g/kg DMI) and CH₄ intensity (g/kg average daily gain; ADG) provide intake- and growth-adjusted metrics that can reduce confounding effects (Rischewski et al. 2017; Coppa et al. 2021). However, selection based solely on CH_4_ production or CH_4_ yield may lead to unintended consequences for growth performance, carcass traits, or efficiency (Smith et al. 2021).

The residual CH₄ emissions (RME) concept has been proposed as a robust phenotypic trait for ranking animals for CH₄ production independent of key growth performance traits (Herd et al. 2014; Bird-Gardiner et al. 2017; Smith et al. 2021). Residual CH₄ emission is calculated as the residual from a regression of CH_4_ production on predictors such as DMI and BW, thereby identifying animals that emit more or less CH₄ than expected for their size and intake. Previous studies in dairy and beef cattle have shown that low-RME animals can produce up to 15–30% less CH_4_ than high-RME counterparts without compromising productivity (Renand et al. 2019; Starsmore et al. 2024; Vargas et al. 2025). Furthermore, RME has been linked to differences in ruminal fermentation profiles (eg volatile fatty acid [VFA] proportions), and the composition of rumen methanogenic communities (Smith et al. 2022; Kim et al. 2024).

Despite growing interest in RME as a selection tool, there is limited information on the broader physiological and behavioral implications of ranking beef cattle by RME, particularly under forage-based feeding systems. Understanding whether phenotypic divergence in RME is associated with differences in growth performance, feed efficiency, feeding behavior, ruminal fermentation patterns, microbiome composition, and nutrient digestibility would provide critical insights into the biological basis of low- CH₄ phenotypes and their potential role in sustainable beef production. Therefore, the objective of this study was to evaluate the influence of phenotypic RME classification on feed intake and efficiency, growth performance, feeding behavior, gas flux, and ruminal fermentation in crossbred beef cattle fed a forage-based diet. We hypothesized that low-RME cattle would exhibit reduced CH_4_ production and CH_4_ per MBW without compromising productivity or efficiency, and that these differences would be accompanied by alterations in feeding behavior, and ruminal fermentation.

## Materials and methods

### Ethic statement

This study was conducted at the Texas A&M University AgriLife Research Center, McGregor, Texas. All animal care and use procedures were approved in accordance with the guidelines for the use of Animals in Agricultural Teaching and Research as approved by the Texas A&M AgriLife Research Institutional Animal Care and Use Committee (IACUC 2024-021A). This experiment was originally designed for a separate objective, nevertheless, the experimental structure and measurements collected were appropriate to support evaluation of the hypotheses and objectives of the present study.

#### Animals and experimental design

A total of 93 clinically healthy crossbred beef cattle representing multiple Angus and *Bos indicus*-influenced breed composites were used in this study. Each animal was fitted with a half-duplex radio frequency identification (RFID) electronic ear tag (Allflex USA Inc., Dallas, TX). Cattle were offered ad libitum access to a sorghum silage-based diet (Table 1), fed twice daily. These animals were subjected to a 4-week adaptation period to acclimate animals to automated feed intake bunks (Vytelle SENSE feed intake measurement system, Lenexa, KS, USA) and GreenFeed Emissions Monitoring System (GEM; C-Lock Inc., Rapid City, SD, USA; Gunter and Beck 2018; French et al. 2025). At the conclusion of the adaptation period, 80 animals (steers = 19; heifers = 61; 529 ± 22 days of age; initial BW = 439.65 ± 61 kg) with consistent GEM visits (≥2 visits/d), each lasting at least 2 mins (French et al. 2025), were enrolled in a 21-d baseline followed by an 84-d experimental phase. These animals were blocked by body weight and sex and then randomly assigned to 1 of 4 pens (20 animals/pen) such that each pen contained both steers and heifers and animals from each body weight block. Each pen was equipped with three automated feed bunks to record daily feed intake and feeding behavior and one GEM to measure daily gas flux of CO_2_, enteric CH_4_, and hydrogen (H_2_) throughout the study. All heifers were administered controlled internal drug-release (CIDR; 1.38 g of P4; Eazi-Breed™ CIDR® Cattle Insert; Zoetis Animal Health) devices to suppress estrous cyclicity and replaced bi-weekly throughout the study period. This was done to minimize behavioral and physiological changes associated with estrus expression that could influence feed intake and activity.

**Table 1:** The ingredient and chemical composition of the sorghum-silage diet used for the 84-d study.

**Ingredient** ^1^	% DM in ration
**Sorghum silage**	23
**Sorghum sudan hay**	17
**Cracked corn**	34
**Dried distillers’ grain**	18
**Molasses**	6
**Mineral premix** ^2^	2

Chemical composition	% of diet DM
**DM, %**	68.2
**CP, %**	15.4
**ADF, %**	28.5
**NDF, %**	43.5
**Ash, %**	9.39
**Ca, %**	1.44
**P, %**	0.44
**TDN, %**	64.4
**ME, Mcal/kg**	2.44
**NEm, Mcal/kg**	1.465
**NEg, Mcal/kg**	0.878

1 Feed ingredients and chemical composition of diet analyzed by Cumberland Valley Analytical Services (CVAS; Waynesboro, PA, USA).

2 Mineral premix contained a minimum of 19.5% Ca, 19.0% NaCl, 0.1% Mg, 0.2% K, 450 ppm Cu, 2800 ppm Zn, 10 ppm Se, 25 ppm I 45,454 IU/kg vit-A, 4091 IU/kg vit-D, 727 IU/kg Vit-E.

DM; Dry Matter, CP; Crude Protein, ADF; Acid Detergent Fiber, NDF; Neutral Detergent Fiber, Ca; Calcium, P; Phosphorus, TDN; Total Digestible Nutrients, ME; Metabolizable Energy, NEm; Net Energy for maintenance, Neg; Net Energy for gain.

### Feed sampling and chemical analysis

Feed samples were collected weekly throughout the study and the samples were frozen at −20°C until analysis. Composite sample was submitted to Cumberland Valley Analytical Services Inc. (CVAS; Waynesboro, PA, USA) for nutrient analysis. Analyses included crude protein (CP; AOAC method 990.03; nitrogen [N] × 6.25; Leco FP-528 Nitrogen Combustion Analyzer, Leco, Joseph, MI, USA), acid detergent fiber (ADF; AOAC method 973.18), neutral detergent fiber (NDF) using heat-stable α-amylase and sodium sulfite (Van Soest et al. 1991), ash (AOAC method 942.05), and a complete mineral panel (AOAC method 985.01). Based on the total digestible nutrients (TDN) of the diet, metabolizable energy (ME) was estimated according to Hales et al. (2022), and net energy values (eg net energy for maintenance, NEm, and net energy for gain, NEg) were estimated using Galyean et al. (2016) prediction equations.

### Rumen fluid sampling and analysis

Approximately 30 mL of rumen fluid were collected on d 0, 7, 28, and 49 from a subset of animals (*n* = 30; steers = 3, heifers = 27) randomly selected from each pen (pen 1, *n* = 8; pen 2, *n* = 8; pen 3, *n* = 7; pen 4, *n* = 7) via esophageal tubing connected to a vacuum pump fitted with an in-line filter. Rumen fluid was collected into a sterile 50 mL conical tube. The first collection was discarded to limit salivary contamination in rumen samples. Samples were then aliquoted into three sterile 15 mL tubes (10 mL × 3) and immediately snap-frozen at the collection site using liquid N_2_. Samples were transported to the laboratory on liquid N_2_ to be stored at −80°C until analysis.

Concentrations (mmol/L) of acetate, propionate, butyrate, valerate, isobutyrate and isovalerate were determined using high-performance liquid chromatography (HPLC; Shimadzu Scientific Instruments, Columbia, MD) as described by Weimer et al. (1991). The system was equipped with a temperature-controlled autosampler (Nexera SIL-30AC UHPLC Cooled Autosampler), a forced-air column oven (CTO-20A), and a UV absorbance detector (SPD-20A UV Detector; all from Shimadzu Scientific Instruments, Columbia, MD). Separation of peaks was achieved using an Aminex HPX-87H column (300 mm × 4.6 mm i.d.; Bio-Rad Laboratories Inc., Hercules, CA) with a corresponding guard column (Bio-Rad Cation H), and detection was performed at 210 nm UV absorbance over a 100 min retention period. For each animal, total VFA was calculated as the sum of all measured VFA, and individual VFA were expressed as a proportion of total VFA. The acetate-to-propionate (A:P) ratio was also calculated.

### Measurement and computation of response variables

The GEM measured the daily CH_4_, CO_2_, and H_2_ emissions of each animal throughout the study and alfalfa pellets (Producers Dehydrated 17 Alfalfa Pellet; Producers Cooperative Association, TX, USA) used as bait. To obtain long duration of visit (≥2 min) each GEM was configured to have 8 maximum drops of alfalfa pellets (∼40 g/drop; every 30 s) for every feeding period, and 5 hours between feeding periods. Carbon dioxide recoveries (100 ± 1.991) were performed every 28 days, air filters were changed once per week or when airflow was less than 26 L/s (Gunter et al. 2017), and auto-calibration performed 3 times per week on each unit throughout the study period.

The automated feed intake system (Vytelle, Lenexa, KS, USA) used in this study to record daily feed intake and feeding behavior have been described in prior publications (Schwartzkopf-Genswein et al. 1999; Mendes et al. 2011). The system is mounted on load cells to record feed disappearance, with an antenna in each bunk detecting the presence of an animal via its unique RFID tag during feeding events. The bunks compute the daily feed intake based on continuous recordings of feed disappearance during feeding events. Assigned feed disappearance (AFD) rates were computed daily for each feed bunk to assess data quality and data were excluded by pen when the AFD of an individual bunk in a pen was less than 90%. The mean (±SD) AFD across the days included in the analysis was 97.3 ± 1%. Feeding behavior traits were derived from bunk visit (BV) events as defined and calculated by Parsons et al. (2020). A BV began when an animal’s RFID tag was first detected at a feed bunk and ended when the tag was no longer detected for more than 100 s, when the tag was detected at a different bunk, or when another animal’s tag was detected at the same bunk (Mendes et al. 2011). Bunk visit frequency was the number of BV events per day, and BV duration was their cumulative daily duration. Eating rate was calculated as a ratio of DMI divided by bunk visit duration.

Prior to morning feeding, body weights were recorded weekly using a Tru-Test scale system (Tru-Test, Mineral Well, TX, USA) and the scale was calibrated prior to weighing animals weekly. Average daily gain (ADG; kg/d) for each animal was estimated as linear regression of body weight (BW; kg) against time (d) over the study period. Initial and final BW were estimated using the regression equation of body weight against d (Koch et al. 1963). Mid-test metabolic BW (MBW; kg ^0.75^) was estimated as the average of initial and final BW raised to the 0.75 power for each animal.

Missing feed intake values were estimated using linear regression of feed intake on study day, as described by Hebart et al. (2004). Daily feed intake for each animal was computed as the average over the 84-d study period to calculate DMI and gain: feed (G:F) ratio was calculated by dividing ADG by DMI. Residual feed intake (RFI) was calculated as the difference between observed dry matter intake (DMI) and the DMI predicted from a linear regression of DMI on ADG and MBW (Koch et al. 1963).

Daily CH_4_ (g/d), CO_2_ (g/d), and H_2_ production (g/d) for each animal were calculated by averaging all valid spot visits (≥40 visits). Methane yield (g/kg DMI) was calculated by dividing CH_4_ production by DMI for each animal. Methane per MBW (g/kg BW^0.75^) were computed for each animal by dividing CH_4_ production by MBW.

The RME was computed for each animal as the residual from the linear regression of CH_4_ production (g/d) on DMI and metabolic BW with original treatment assignment included as a fixed effect. Animals were ranked by RME and classified into one of three RME phenotypic groups according to Smith et al. (2021): low (RME < 0.5 SD below the average), medium (RME below and above the average), or high (RME > 0.5 SD above average).

#### Statistical analysis

For all statistical analysis, data was tested for normality using the Shapiro–Wilk test (W ≥ 0.90). Homogeneity of variance was assessed using Levene’s test. Statistical significance was set at *P* ≤ 0.05, and tendencies were considered when 0.10 > *P* ≥ 0.05. Tests for outliers were performed using 3 SD below and above the average for each variable. One heifer died during the first week of the study and another was isolated for treatment due to ill-health, therefore these animals were excluded from analysis. Gas flux data were excluded when the length of the visit was less than 2 min, and the airflow was less than 26 L/s (Arthur et al. 2017; Gunter and Beck 2018). Each animal met the recommended visits of ≥ 40 spot visits to GEM with ≥ 2 mins duration (Dressler et al. 2023; Beck et al. 2024). Hydrogen emissions were log-transformed prior to analysis to improve normality. Results are presented as back-transformed least squares means for interpretability.

To assess the association of divergent RME phenotypes on growth performance, feed intake, feed efficiency, feeding behavior, and gas flux, a linear mixed model was fitted using JMP (version 9.4; SAS Institute Inc., Cary, NC, USA). The model included fixed effects of RME ranking and sex and pen as a random effect.

For the analysis of rumen fermentation variables, repeated measure analysis was completed with a model including the fixed effect of RME, day and their interaction, sex and pen were used as random effects, animal was specified as the subject of the repeated measures, and an unstructured covariance structure was used to model the repeated observations. Results were reported as least-square means and separated using the Tukey HSD. Correlation analyses were determined in JMP using the multivariate method and the Pearson’s correlations coefficient (r) reported and was considered very strong if ≥ 0.80, strong if < 0.80 and ≥ 0.60, moderate if < 0.60 and ≥ 0.40, and weak < 0.40 and ≥ 0.10 (Akoglu 2018).

## Results

### Animal feed intake, growth performance, and feed efficiency

Table 2 displays the effects of RME classification on intake, growth performance, and feed efficiency over the 84-d study. Animals classified as low, medium and high differed in RME classification (*P* < 0.01), with low RME animals being CH_4_ efficient (−22.15 g/d), medium RME animals being CH_4_ intermediate (0.62 g/d) and high RME animals being CH_4_ inefficient (20.17 g/d). The classification of RME did not affect daily DMI, initial BW, final BW, ADG, and mid-test metabolic BW (*P* ≥ 0.10). However, a tendency (*P* = 0.08) for lower G:F was observed in low RME compared with medium and high RME. Residual feed intake (RFI), although was not affected by RME classification (*P* = 0.31), animals in high RME had numerically lower RFI values than animals in low and medium RME groups.

**Table 2:** Effect of animals with divergent residual methane emission on intake, growth performance, and feeding efficiency of animals fed sorghum silage-based diet.

	RME classification				
*Variable*	Low	Medium	High	SE	***P*-value** ^1^
**n**	25	27	26	–	–
**Residual CH_4_, g/d**	−22.15^c^	0.62^b^	20.17^a^	2.34	<0.001
***Feed intake***					
** DMI, kg/d**	9.22	9.07	9.18	0.57	0.940
***Growth performance traits***					
** Initial BW, kg**	501.4	472.6	482.9	44.5	0.101
** Final BW, kg**	541.1	532.8	541.3	54.7	0.803
** ADG, kg/d**	0.60	0.69	0.70	0.08	0.389
** Metabolic BW, kg^0.75^**	108.5	106.1	107.6	7.7	0.504
***Feed efficiency traits***					
** G:F**	0.054	0.074	0.073	0.012	0.082
** RFI, kg/d**	0.413	0.003	−0.023	0.466	0.308

1 *P*-value threshold for significance is *P*  ≤  0.05.

a-c Rows not connected by the same superscripts are significantly different. CH_4_; Methane, DMI; Dry Matter Intake, BW; Body Weight, ADG; Average Daily Gain, G:F; ratio of ADG to DMI, RFI; Residual Feed Intake.

### Animal bunk visit feeding behavior traits

Bunk visit duration, head down duration, and eating rate were not affected by RME classification (*P* ≥ 0.80). However, low RME tended to have fewer bunk visit events compared to their medium and high counterparts (*P* = 0.08) as presented in Table 3.

**Table 3:** Effect of animals with divergent residual methane emission on feeding behavior of animals fed sorghum silage-based diet.

	RME Classification				
*Variable*	Low	Medium	High	SE	*P*-value^1^
**n**	25	27	26	–	–
***Bunk visit (BV) behavior traits***					
** BV frequency, events/d**	51	60	58	10	0.085
** BV duration, min/d**	109.5	110.6	108.3	4.1	0.943
** Head down duration, min/d**	58.5	61.1	57.4	3.5	0.803
** BV eating rate, g/min**	87.0	85.4	87.3	5.8	0.900

1 *P*-value threshold for significance is *P*  ≤  0.05.

BV; Bunk Visit, RME; Residual Methane Emission.

### Animal gas emissions

During the 84-day study period, the total number of animal visits to the GEM was 191 ± 34 (mean ± SD) which corresponds to a mean of 2.28 ± 0.407 visits per day. Table 4 displays the comparison of GEM traits and gas flux traits between low, medium and high RME groups. Frequency of visits to GEM, GEM visit per day, GEM airflow rate and GEM visit duration were not affected by RME classification during the study period (*P* ≥ 0.44).

**Table 4:** Effect of animals with divergent residual methane on gaseous emissions of animals fed sorghum silage-based diet.

RME classification					
*Variable*	Low	Medium	High	SE	***P*-value** ^2^
**n**	25	27	26	–	–
^1^ ***GEM traits***					
** GEM frequency per 84-d**	191	187	198	6	0.480
** GEM visit per day**	2.28	2.22	2.36	0.07	0.481
** GEM airflow rate, L/s**	38.3	38.2	38.3	0.5	0.441
** GEM visit duration, min/event**	4.42	4.44	4.51	0.15	0.594
***Gas flux traits***					
** CH_4_ production, g/d**	163.52^c^	184.55^b^	205.26^a^	8.27	<0.001
** CH_4_ yield, g/kg DMI**	17.86^c^	20.29^b^	22.36^a^	1.03	<0.001
** CH_4_ per MBW, g/kg BW ^0.75^**	1.51^c^	1.75^b^	1.92^a^	0.05	<0.001
** CO_2_ production, g/d**	6859^b^	7019^b^	7446^a^	381	0.002
** Log H_2_ production, g/d**	0.512	0.516	0.593	0.043	0.062

1 GEM; Gaseous Emission Monitoring, CH_4_; Methane, CO_2_; Carbon Dioxide, H_2_; Hydrogen, DMI; Dry Matter Intake, BW; Body Weight, MBW; Metabolic Body Weight, RME; Residual Methane Emission.

2 *P*-value threshold for significance is *P*  ≤  0.05.

a-c Rows not connected by the same superscripts are significantly different.

The low RME animals had 20% less CH_4_ production (g/d; *P* < 0.01), 20% less CH_4_ yield (g/kg DMI; *P* < 0.01), 21% less CH_4_ per MBW (g/kg BW ^0.75^; *P* < 0.01) compared with the high RME animals. Carbon dioxide production was 8% lower in the low RME animals (g/d; *P* < 0.01) than high RME animals. Hydrogen production tended to be 14% lower in low RME animals compared to high RME animals (g/d; *P* = 0.06).

### Rumen fermentation characteristics

Comparison of ruminal fermentation molar proportions among low, medium and high RME groups is presented in Table 5. No differences were observed between low, medium and high RME in VFA molar proportions. A day effect was observed on proportion of acetate, butyrate, and isovalerate (*P* < 0.01), however, RME x day interaction was not significant (*P* > 0.27). Figure 1 shows the VFA concentrations (mmol/L) across the experimental days.

**Figure 1:**
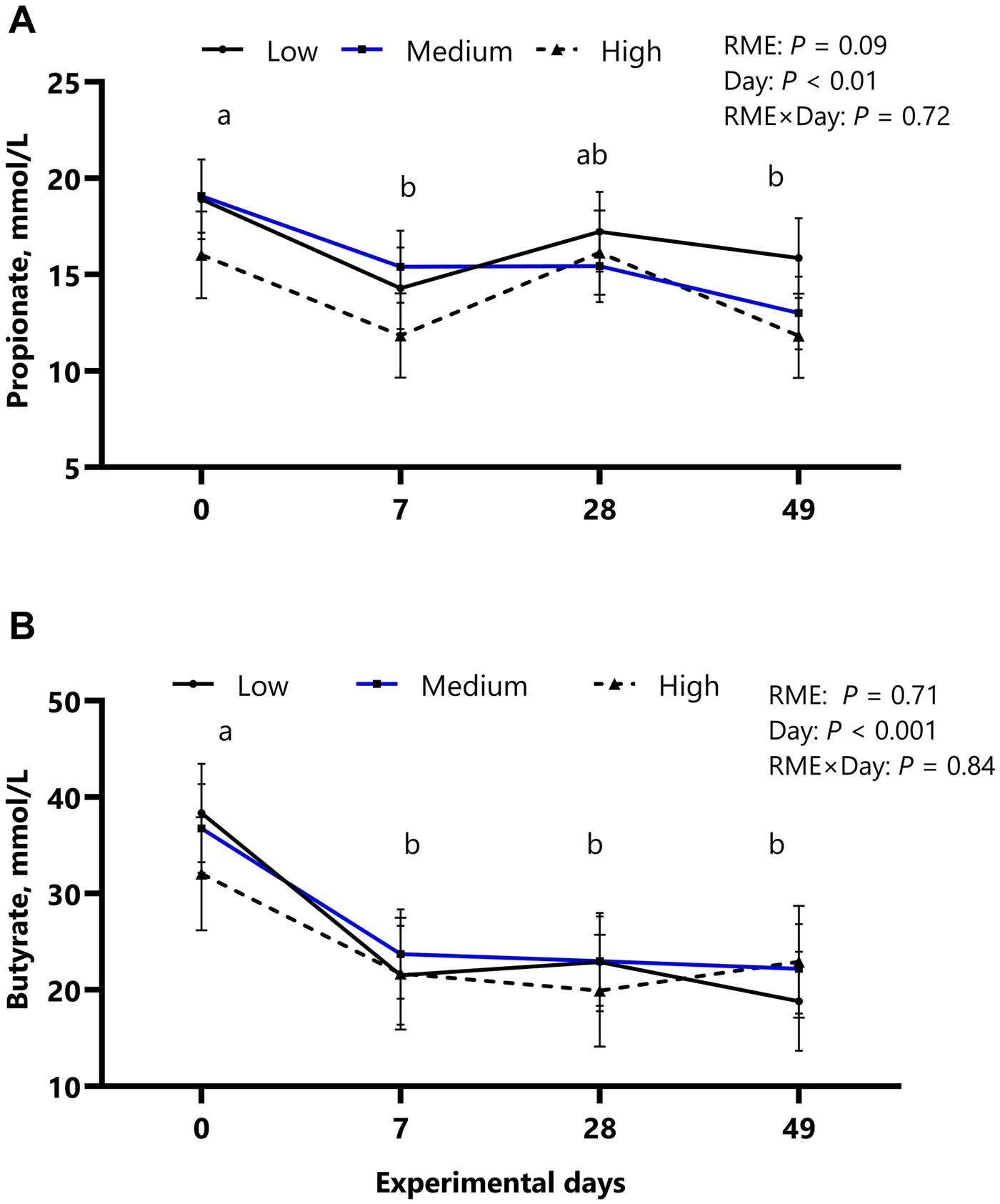
Temporal changes in volatile fatty acid concentrations (VFA, mmol/L) of propionate (A) and butyrate (B) in animals (*n* = 30) with divergent residual methane emissions. Low: efficient animals (*n* = 9). Medium = moderate animals (*n* = 11). High: inefficient animals (*n* = 10). ^a-b^ Days not connected by the same superscripts are significantly different. RME; residual methane emission.

**Table 5:** Effect of animals with divergent methane emission on ruminal fermentation.

	RME classification				*P*-value^1^		
*Proportion, %^2^*	Low	Medium	High	SE	RME	Day	RME x D
**n**	9	11	10	–	–	–	–
**Acetate**	54.31	54.78	52.91	2.39	0.463	<0.001	0.273
**Propionate**	16.28	15.23	15.24	0.91	0.190	0.168	0.506
**Butyrate**	26.35	26.95	29.11	2.98	0.352	0.001	0.284
**Valerate**	0.63	0.66	0.46	0.20	0.295	0.195	0.846
**Isobutyrate**	0.75	0.66	0.69	0.06	0.229	0.109	0.886
**Isovalerate**	1.78	1.60	1.62	0.22	0.205	0.012	0.698
**Total VFA, mmol/L**	104.19	105.00	94.17	3.88	0.132	0.005	0.926
**A:P**	3.46	3.71	3.62	0.08	0.135	<0.001	0.732

1 *P*-value threshold for significance is *P*  ≤  0.05.

2 Proportion of volatile fatty acid in percentages calculated as the concentration of each VFA/total VFA concentration multiplied by 100.

RME; Residual Methane Emission, VFA; Volatile Fatty Acid, A:P; ratio of Acetate to Propionate.

### Association of gaseous emission variables

Table 6 presents the Pearson correlation coefficients of CH_4_ variables. Methane production was strongly correlated with CH_4_ per MBW (r = 0.76) and CO_2_ production (r = 0.78) and moderately correlated with H_2_ production (r = 0.44). Methane yield was weakly correlated with CH_4_ per MBW (r = 0.28), while CH_4_ per MBW was weakly correlated with CO_2_ production (r = 0.30), H_2_ production (r = 0.38), GEM airflow rate (r = 0.25), and GEM visit duration (r = 0.26). Carbon dioxide production was weakly correlated with H_2_ production (r = 0.34).

**Table 6:** Correlation coefficients (r) of gaseous emissions variables.

	CH_4_ prod.	CH_4_ yield	CH_4_ per MBW	CO_2_, prod.	H_2_ prod.	GEM airflow	GEM visit duration
**CH_4_ production, g/d**	–	0.18	0.76*	0.78*	0.44*	0.15	0.17
**CH_4_ yield, g/kg DMI**	–	–	0.29*	−0.07	−0.02	−0.19	0.08
**CH_4_ per MBW, g/kg BW ^0.75^**	–	–	–	0.30*	0.38*	0.25*	0.26*
**CO_2_ production, g/d**	–	–	–	–	0.34*	−0.03	−0.03

* Statistically significant at *P*-value < 0.05.

GEM; Gaseous Emission Monitoring, GEM airflow; Gaseous Emission Monitoring airflow (L/s), GEM duration; Gaseous Emission Monitoring duration of visit (event/min), CH_4_; Methane production (g/d), CO_2_; Carbon Dioxide production (g/d), H_2_; Hydrogen production (g/d), DMI; Dry Matter Intake, BW; Body Weight, MBW; Metabolic Body Weight, CH_4_ yield; Methane yield (g/kg DMI), CH_4_ per MBW (g/kg BW ^0.75^).

### Associations of intake, growth performance and feed efficiency with gaseous emission variables

Table 7 presents the Pearson correlation coefficients for DMI, ADG, feed efficiency and gaseous emissions. Dry matter intake was moderately correlated with daily CH_4_ production (r = 0.54), weakly correlated with CH_4_ per MBW (r = 0.25) and H_2_ production (r = 0.31), strongly correlated with CO_2_ production (r = 0.62), and strongly negatively correlated with CH_4_ yield (r = −0.71). Average daily gain was moderately correlated with CH_4_ production (r = 0.52), CH_4_ per MBW (r = 0.31), CO_2_ production (r = 0.48), and H_2_ production (r = 0.39), strongly correlated with DMI (r = 0.70), and moderately negatively correlated with CH_4_ yield (r = −0.41). Metabolic BW was moderately correlated with daily CH4 production (r = 0.46) and DMI (r = 0.48), strongly correlated with CO_2_ production (r = 0.75), and weakly negatively correlated with CH4 per MBW (r = −0.23). Residual CH4 was very strongly correlated with CH4 production (r = 0.81) and CH4 per MBW (r = 0.88), strongly correlated with CH4 yield (r = 0.63), and weakly correlated with CO_2_ production (r = 0.39) and H_2_ production (r = 0.33). Feed efficiency was moderately correlated with daily CH_4_ production (r = 0.44) and strongly correlated with H_2_ production (r = 0.53), while weak correlations were observed with CH_4_ per MBW (r = 0.34), CO_2_ production (r = 0.30), DMI (r = 0.39), and RME (r = 0.26). The relationship of RME, with CH_4_ production, CH_4_ yield, G:F and RFI are displayed Fig. 2.

**Figure 2:**
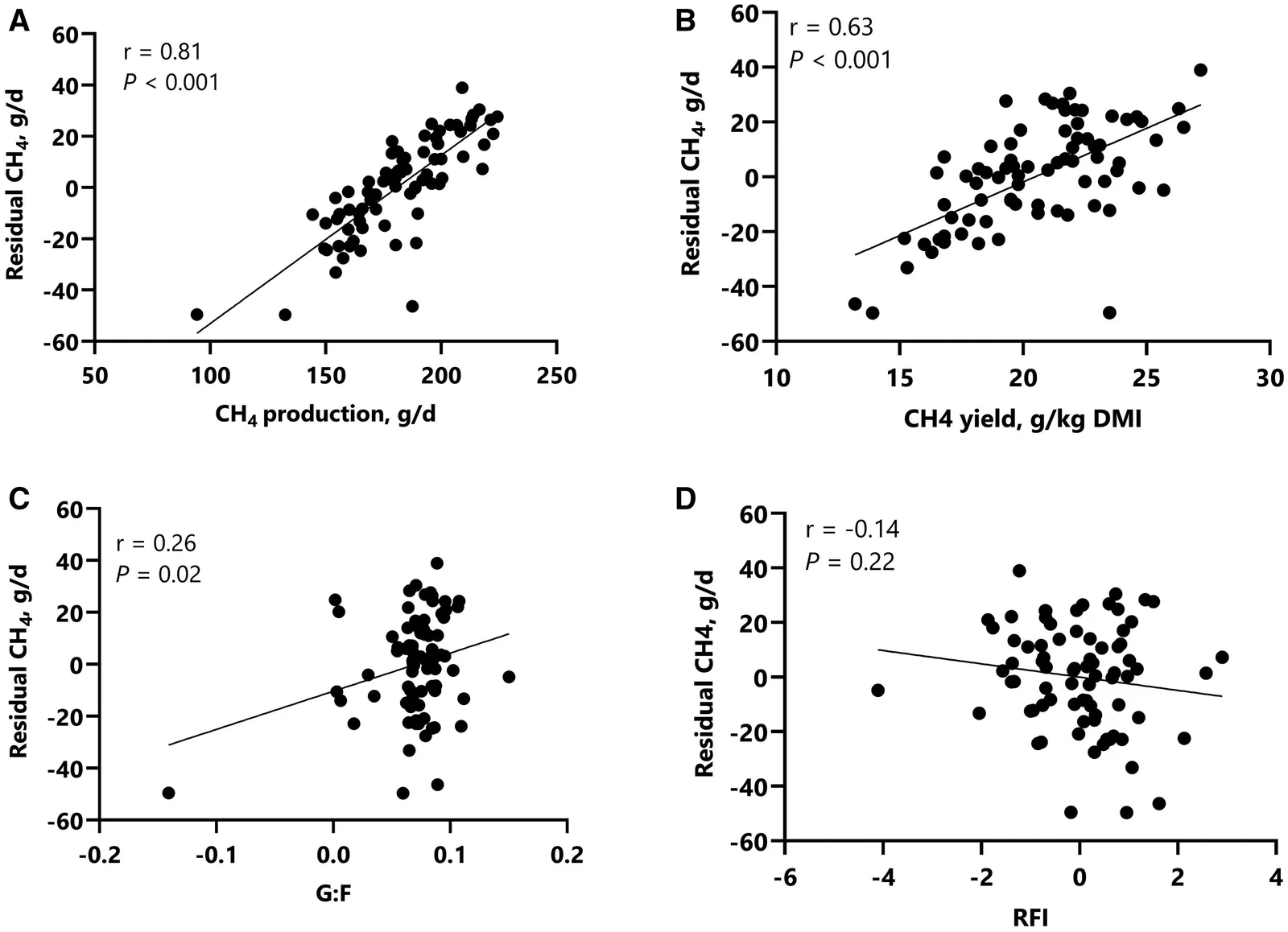
The association of residual methane emission (RME) with methane production (A; CH_4_, g/d; r = 0.81; *P* < 0.001), methane yield (B; CH_4_ yield, g/kg DMI; r = 0.63; *P* < 0.001), gain-to-feed ratio (C; G:F, kg/d; r = 0.26; *P* = 0.02), and residual feed intake (D; RFI, kg/d; r = −0.14; *P* = 0.22).

**Table 7:** Correlation coefficients (r) of gaseous emissions variables, growth performance, feed efficiency.

	DMI	ADG	Initial	Final BW	MBW	Residual CH_4_	RFI	G:F
**CH_4_ production, g/d**	0.54*	0.52*	0.39*	0.49*	0.46*	0.81*	0.16	0.44*
**CH_4_ yield, g/kg DMI**	−0.71*	−0.41*	−0.09	−0.15	−0.19	0.63*	−0.64*	−0.15
**CH_4_ per MBW, g/kg BW^0.75^**	0.25*	0.31*	−0.29*	−0.17	−0.23*	0.88*	0.18	0.34*
**CO_2_ production, g/d**	0.62*	0.48*	0.70*	0.77*	0.75*	0.39*	0.22	0.30*
**H_2_ production, g/d**	0.31*	0.39*	0.12	0.22	0.18	0.33*	0.04	0.53*
**DMI, kg/d**	–	0.70*	0.40*	0.52*	0.48*	–	0.67*	0.39*
**ADG, kg/d**	–	–	0.23*	0.50*	0.39*	0.15	–	0.89*
**Initial BW, kg**	–	–	–	0.92*	0.98*	−0.04	0.04	0.07
**Final BW, kg**	–	–	–	–	0.99*	0.03	−0.04	0.35*
**Metabolic BW, kg^0.75^**	–	–	–	–	–	–	–	0.23*
**Residual CH_4_, g/d**	–	–	–	–	–	–	−0.14	0.26*
**RFI, kg/d**	–	–	–	–	–	–	–	−0.29*

* Statistically significant at *P*-value < 0.05.

GEM; Gaseous Emission Monitoring, CH_4_; Methane production (g/d), CO_2_; Carbon Dioxide production (g/d), H_2_; Hydrogen production (g/d), DMI; Dry Matter Intake (kg/d), BW; Body Weight, ADG; Average Daily Gain (kg/d), MBW; Metabolic Body Weight (kg^0.75^), G:F; ratio of ADG to DMI, RFI; Residual Feed Intake (kg/d).

### Associations of bunk visit behaviors with gaseous emission, intake, growth performance and feed efficiency

Pearson correlation coefficients for BV frequency, BV duration and head down duration (HDD) with intake, growth performance, feed efficiency and gaseous emissions are displayed in Table 8. Bunk visit frequency was weakly correlated with CH_4_ per MBW (r = 0.38) and RFI (r = 0.35). Bunk visit duration was weakly correlated with CH_4_ production (r = 0.29) and CO_2_ production (r = 0.35), moderately correlated with DMI (r = 0.56), ADG (r = 0.59), and G:F (r = 0.46), and moderately negatively correlated with CH_4_ yield (r = −0.41). Head down duration was weakly correlated with CH_4_ production (r = 0.27) and CO_2_ production (r = 0.31), moderately correlated with DMI (r = 0.55) and ADG (r = 0.44), and moderately negatively correlated with CH_4_ yield (r = −0.42). Eating rate was weakly positively correlated with DMI (r = 0.31) and RFI (r = 0.39).

**Table 8:** Correlation coefficients (r) of gaseous emissions variables, growth performance, feed efficiency and bunk visit behaviors.

	BVF, event/d	BVD, min/d	HDD, min/d	Eating rate, g/min
**CH_4_ production, g/d**	0.07	0.29*	0.27*	0.21
**CH_4_ yield, g/kg DMI**	−0.21	−0.41*	−0.42*	−0.19
**CH_4_ per MBW, g/kg BW^0.75^**	0.38*	0.17	0.13	0.08
**CO_2_ production, g/d**	−0.09	0.35*	0.31*	0.19
**H_2_ production, g/d**	0.08	0.19	0.02	0.10
**DMI, kg/d**	0.18	0.56*	0.55*	0.31*
**ADG, kg/d**	0.08	0.59*	0.44*	0.01
**Initial BW, kg**	−0.44*	0.16	0.20	0.19
**Final BW, kg**	−0.39*	0.31*	0.29*	0.15
**Metabolic BW, kg^0.75^**	−0.42*	0.25*	0.25*	0.18
**Residual CH_4_, g/d**	0.13	−0.001	−0.03	0.04
**RFI, kg/d**	0.35*	0.195	0.33*	0.39*
**G:F**	0.05	0.46*	0.26*	−0.10

* Statistically significant at *P*-value < 0.05.

BVF; Bunk Visit Frequency, BVD; Bunk Visit Duration, HDD; Head Down Duration, GEM; Gaseous Emission Monitoring, CH_4_; Methane, CO_2_; Carbon Dioxide, H_2_; Hydrogen, DMI; Dry Matter Intake, BW; Body Weight, MBW; Metabolic Body Weight, ADG; Average Daily Gain, G:F; ratio of ADG to DMI, RFI; Residual Feed Intake.

## Discussion

Our hypothesis for this study was that low-RME cattle would exhibit reduced CH₄ emissions without compromising productivity or efficiency, and that these differences would be accompanied by changes in feeding behavior and ruminal fermentation. Residual CH₄ emission did not influence intake and growth performance indices such as DMI, initial BW, final BW, ADG, and MBW. This may indicate that methane classification captured variation in emissions rather than variation in productivity. From a production standpoint, this is encouraging because it suggests that reducing methane emissions may be achievable without negatively affecting animal performance. The low RME group demonstrated a tendency towards decreased feed efficiency, as evidenced by a relatively low gain-to-feed ratio compared to medium and high RME class. One possible explanation is that energy conserved from reduced CH_4_ production in this animal population may have been redirected to maintenance requirements, tissue turnover, immune function, or other metabolic functions rather than being translated into additional weight gain. Contrary to the findings by Smith et al. (2021) who reported no difference in feed efficiency traits between low and high RME animals. The discrepancy may, in part, be attributable to differences in dietary composition between studies. Cattle in the present study were fed a higher-forage diet whereas Smith et al. (2021) used a lower-fiber, higher-energy diet. Because dietary fiber concentration influences ruminal fermentation patterns, nutrient digestibility, and energy availability, differences in diet composition may have contributed to the contrasting relationships between RME and feed efficiency observed across studies. The lack of difference in DMI observed between RME groups in the present study was reported in other studies investigation RME such as Denninger et al. (2019) in lactating dairy cows and Vargas et al. (2025) in beef cattle. Collectively, these findings indicate that reductions in CH_4_ emissions can occur independently of feed intake and growth performance, supporting the concept that intrinsic biological variation drives CH_4_ efficiency in beef cattle. The potential reduction in feed efficiency observed in low RME cattle warrants further investigation to confirm its biological relevance and consistency across production conditions.

Behavioral metrics related to feeding, such as bunk visit duration and head-down duration, were not significantly affected by RME classification in the present study. Nevertheless, the low RME group tended to exhibit a decrease in bunk visit events compared with medium RME. Fewer bunk visit events have been previously reported to be associated with efficient animals (Parsons et al. 2020; O’Reilly et al. 2024). Furthermore, efforts have been directed toward identifying practical biomarkers that could reduce the need for direct measurement of CH_4_ emissions in individual animals, given the expense and logistical challenges of CH_4_ quantification on farm (Negussie et al. 2017). One proposed indicator is eating rate, under the premise that faster consumption would shorten rumen retention time of feed in the rumen and thereby lower CH_4_ yield (Pickering et al. 2015). Beauchemin et al. (2022) determined no association between eating rate and CH_4_ emission. Similarly, in the present study, eating rate was moderately associated with DMI but was not significantly associated with RME. These findings suggest that although eating behavior may influence feed intake dynamics, it does not appear to be a reliable predictor distinguishing between low- and high- CH₄ emitting cattle.

The GEM system is an automated, noninvasive method that has been used in enteric CH₄ emissions research; however, its accuracy can be influenced by the frequency and consistency of animal visits because daily GEM emission estimates are derived from repeated short-duration visits. Reliable estimates depend on how well GEM visits encompass the day and the extent of diurnal variation of the emissions (Beck et al. 2025). In the present study, cattle visited the GEM unit an average of 2.28 ± 0.41 visits/d, which was within the range reported by Hammond et al. (2015; 2.34 ± 1.05 visits/d), Denninger et al. (2019; 2.52 ± 0.43 visits/d). This frequency, together with adequate temporal distribution of visits throughout the day, supports the reliability of the GEM-derived methane estimates.

Methane is produced as a by-product of ruminal microbial fermentation with methanogens utilizing H₂ and CO₂ as substrates as the major pathway to methanogenesis (Leahy et al. 2013; Roehe et al. 2016). In this study, the classification of animals according to RME resulted in 8% reduced CO_2_ production and a tendency towards a 14% lower H_2_ production in the low RME group than their high counterparts possibly indicate altered fermentation dynamics and energy partitioning within the rumen. However, these gas fluxes should not be interpreted as direct evidence of improved metabolic efficiency on their own, because reduced gas production may also arise from lower fermentative activity or shifts in microbial metabolism. Alternative explanations, including compensatory digestion or absorption in the lower gut, cannot be excluded and would require direct measurements of microbial populations and post-ruminal nutrient use to confirm the mechanism. These observations are consistent with those of Smith et al. (2021), who reported lower CO_2_ production, reduced predicted H_2_ production based on VFA profiles, and a greater molar proportion of propionate, which suggests diversion of H_2_ toward propionate formation in low-RME finishing beef cattle. Similarly, Vargas et al. (2025) reported reduced CO₂ emissions in low RME cattle fed a backgrounding diet. The absence of differences in DMI and growth performance in the present study suggests that the observed gas reductions were not associated with reduced productive output but rather may reflect differences in how dietary energy was utilized.

The quantification of enteric CH_4_ production in the present study revealed pronounced differences across RME classifications, with the low RME group producing 20% less CH_4_ production compared with their high RME counterparts. Similarly, low RME group resulted in 20% less CH_4_ yield and 21% less CH_4_ per MBW than the high RME group. Similar to the present study, Vargas et al. (2025) classified steers into low and high RME during both the backgrounding and finishing phases, reporting lower CH_4_ production in low RME animals compared to high emitters. The authors further recommended that RME classification be conducted separately within each production phase, as animals may re-rank in response to dietary changes. Such shifts are likely driven by alterations in physiological characteristics, feeding behavior and the ruminal microbiome, both of which can influence CH_4_ production. Smith et al. (2021) reported similar results on CH_4_ emissions of finishing beef cattle with low emitters producing lower CH₄ production (g/d), CH₄ yield (g/kg DMI) and CH₄ intensity (g/kg ADG) than their high emitting counterparts. The reduction observed across all CH₄ traits in the low RME class in their study occurred without compromising animal performance, reinforcing the value of RME as a criterion for identifying cattle that are genuinely divergent in CH₄ emissions independent of productivity and this highlights RME as a selection tool for reducing emissions while safeguarding profitability (Herd et al. 2014; Beauchemin et al. 2020; Smith et al. 2021).

## Conclusion

In summary, the results indicate that while the classification based on RME did not affect growth performance parameters, rumen fermentation profiles, although low RME animals exhibited ∼20% lower CH₄ production. The tendency for reduced feed efficiency observed in low-RME animals highlights the need for further evaluation of this trait across different production systems. Further investigation into mechanistic pathways, genetic underpinnings, and interactions of microbiome composition of low RME will be critical for translating these insights into practical applications for the livestock industry. The overall goal is to develop ruminant production systems that are more efficient while minimizing their environmental footprint.

## List of abbreviations

A:P: Acetate-to-propionate ratio
ADF: Acid detergent fiber
ADG: Average daily gain
AFD: Assigned feed disappearance
BV: Bunk visit
BVD: Bunk visit duration
BVF: Bunk visit frequency
BW: Body weight
Ca: Calcium
CH_4_: Methane
CIDR: Controlled internal drug-release
CO_2_: Carbon dioxide
CP: Crude protein
Cu: Cupper
d: Day
DM: Dry matter
DMI: Dry matter intake
G:F: Gain-to-feed ratio
GEM: Gaseous emission monitoring
H_2_: Hydrogen
HDD: Head down duration
HPLC: High-performance liquid chromatography
K: Potassium
MBW: Metabolic body weight
ME: Metabolizable energy
Mg: Magnesium
N_2_: Nitrogen
NaCl: Sodium chloride
NDF: Neutral detergent fiber
NEg: Net energy for growth
NEm: Net energy for maintenance
P: Phosphorus
RFI: Residual feed intake
RFID: Radio frequently identification
RME: Residual methane emission
SD: Standard deviation
Se: Selenium
TDN: Total digestibility nutrient
VFA: Volatile fatty acid
Vit-A: Vitamin A
Vit-D: Vitamin D
Vit-E: Vitamin E
Zn: Zinc
